# Capnocytophaga canimorsus-Induced Sepsis Unmasking an Aortic Aneurysm: A Case Report

**DOI:** 10.7759/cureus.107986

**Published:** 2026-04-29

**Authors:** Marta Barrigas, Catarina Coelho, Mariana Certal, Bruna Malheiro, Neusa Lages, Beatriz Exposito

**Affiliations:** 1 Internal Medicine, Unidade Local de Saúde de Trás-os-Montes e Alto Douro, Chaves, PRT; 2 Clinical Pathology, Unidade Local de Saúde de Trás-os-Montes e Alto Douro, Vila Real, PRT; 3 Anesthesiology, Unidade Local de Saúde de Santo António, Porto, PRT

**Keywords:** aortic aneurysm, asplenia, bacteremia, capnocytophaga canimorsus, disseminated intravascular coagulation, sepsis

## Abstract

*Capnocytophaga canimorsus* is a gram-negative, anaerobic bacillus commonly found in the oral flora of dogs and cats. While often transmitted through animal bites or scratches, severe infections can occur even without documented exposure, particularly in immunocompromised individuals such as asplenic patients. Although rare, mycotic aneurysms caused by *C. canimorsus* are associated with high mortality.

We report the case of a 53-year-old male with a history of splenectomy due to diffuse large B-cell lymphoma, currently in remission, who presented with malaise, fever, dyspnea, and abdominal pain. He rapidly progressed to septic shock and multiorgan failure, including coagulopathy consistent with disseminated intravascular coagulation (DIC). Despite broad-spectrum antibiotics and intensive support, the patient died within hours of admission. Blood cultures later confirmed *C. canimorsus* bacteremia. Postmortem examination revealed a ruptured aortic aneurysm.

This case highlights the fulminant progression of *C. canimorsus* infection in an asplenic patient, culminating in a fatal aortic aneurysm rupture. Notably, there was no history of animal exposure, reinforcing the need for high clinical suspicion, especially in high-risk populations. Although empiric therapy with beta-lactam/beta-lactamase inhibitors is recommended, early recognition and possible surgical intervention remain crucial for survival. Infections caused by *C. canimorsus* should be considered in septic patients with risk factors such as splenectomy, even in the absence of animal contact. Rapid deterioration and rare complications, like aortic aneurysm rupture, underscore the importance of early diagnosis and aggressive multidisciplinary management.

## Introduction

*Capnocytophaga canimorsus* is an anaerobic, gram-negative bacillus that exists as part of the normal oral flora of dogs and cats. It can cause infection in both animals and humans, especially in those who have risk factors such as asplenia, alcohol abuse, and immunosuppression [1].

Symptoms of infection may include blisters, redness, and swelling at the site of a bite or scratch wound; fever; diarrhea and/or stomach pain; vomiting; and headache and/or confusion. However, the disease can progress rapidly from a mild, localized infection to systemic infection, sepsis, and death. Mortality is usually due to complications from shock, disseminated intravascular coagulation (DIC), and organ failure. In rare cases, severe bacteremia may lead to vascular complications, including the development or destabilization of mycotic aneurysms. These complications have been described in the literature but remain uncommon and are not fully characterized.

In the context of the present case, the possible association between infection and aneurysm instability is considered based on clinical, analytical, and autopsy findings. The presentation of rupture of an aortic aneurysm in a septic patient is rare. It typically occurs in the setting of advanced sepsis and may be difficult to recognize due to its nonspecific presentation and the rapid clinical deterioration of affected patients. Diagnostic challenges arise from the fastidious nature of the organism, which can delay microbiological identification, as well as from the overlap between septic and vascular symptoms. As a result, early diagnosis requires a high index of suspicion, particularly in patients with risk factors and a history of animal exposure [1-4].

## Case presentation

A 53-year-old male with a prior history of diffuse large B-cell non-Hodgkin lymphoma in remission and splenectomy, and no documented history of aortic aneurysm, presented to the Emergency Department with a 24-hour history of malaise, myalgias, and fever, associated with dyspnea and progressively worsening colicky abdominal pain. He rated the pain intensity as 9 on the 0-10 numerical rating scale (NRS) and denied exacerbating or relieving factors. He denied recent animal exposure, including dog or cat bites or scratches, as well as any oral manipulation. His vaccination status was not up to date.

On initial examination, the patient was alert and fully oriented, although visibly anxious and restless. Vital signs were notable for a tympanic temperature of 39°C, heart rate of 123 beats per minute, respiratory rate of 30 breaths per minute, blood pressure of 150/100 mmHg, and oxygen saturation of 98% on room air (fraction of inspired oxygen of 21%). Peripheral examination revealed signs of hypoperfusion, including marked cutaneous mottling, most pronounced over the lower limbs and abdominal wall. Cardiac auscultation demonstrated a regular rhythm with marked tachycardia. Pulmonary auscultation revealed bilaterally diminished breath sounds. Abdominal examination showed diffuse tenderness on palpation, without rebound or guarding, and preserved bowel sounds.

**Figure 1:**
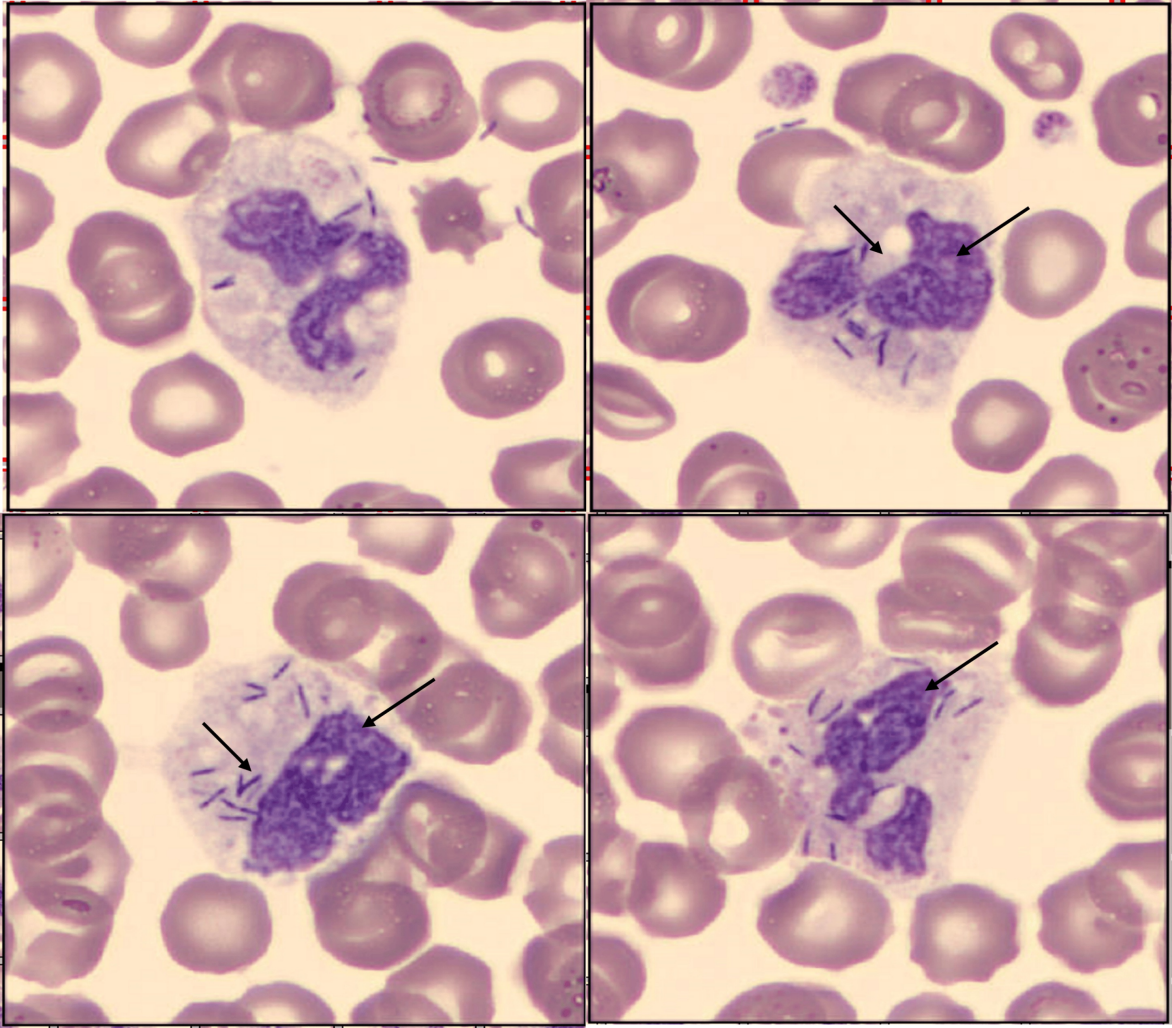
Peripheral blood smear Peripheral blood smear stained with May-Grünwald-Giemsa showed neutrophils containing intracellular bacillary structures, subsequently identified by blood culture as *Capnocytophaga canimorsus*. Images were digitized using CellaVision® software (CellaVision AB, Lund, Sweden).

Initial laboratory evaluation revealed metabolic acidosis with hyperlactatemia (pH 7.38, pO₂ 90 mmHg, pCO₂ 20 mmHg, HCO₃⁻ 21.4 mEq/L, lactate 7.0 mmol/L). Renal dysfunction was evident, with elevated serum creatinine (2.30 mg/dL) and blood urea nitrogen (49 mg/dL). Serum electrolytes - sodium, potassium, calcium, and chloride - were within the normal range. Coagulation studies demonstrated marked coagulopathy, with an international normalized ratio (INR) of 2.48, activated partial thromboplastin time (APTT) of 135 seconds, fibrinogen of 130 mg/dL, and markedly elevated D-dimer (>20.0 μg/mL), consistent with DIC. C-reactive protein was elevated (15.12 mg/dL). Complete blood count showed a normal hemoglobin level (15.6 g/dL), mean corpuscular volume (MCV) 97.5 fL, and mean corpuscular hemoglobin (MCH) 29 pg, but thrombocytopenia (87 × 10⁹/L) and leukopenia (3.95 × 10⁹/L), with neutrophil predominance (89.1%) and lymphopenia (9.1%). Peripheral blood smear revealed a left shift in granulocytic maturation, hyposegmented neutrophils with cytoplasmic vacuolization, and neutrophils actively phagocytosing bacillary forms (Figure 1).

Additional laboratory tests showed total protein of 6.2 g/dL, serum albumin of 4.0 g/dL, and total bilirubin of 1.00 mg/dL. Liver function tests revealed mildly elevated gamma-glutamyl transferase (GGT, 58 U/L), with normal levels of aspartate aminotransferase (AST), alanine aminotransferase (ALT), alkaline phosphatase, and lactate dehydrogenase. Cardiac biomarkers showed elevated N-terminal pro-B-type natriuretic peptide (NT-proBNP, 5954 pg/mL), with normal troponin I (0.028 ng/mL), and urinalysis was unremarkable (Table 1).

**Table 1:** Laboratory results INR, International Normalized Ratio; APTT, Activated Partial Thromboplastin Time; MCV, Mean Corpuscular Volume; GGT, Gamma-Glutamyl Transferase; AST, Aspartate Aminotransferase; ALT, Alanine Aminotransferase; NT-proBNP, N-terminal pro-B-type Natriuretic Peptide

Analytical parameter	Result	Reference range
pH	7.38	7.35-7.45
pO₂, mmHg	90	80-100
pCO₂, mmHg	20	35-45
HCO₃, mEq/L	21.4	22-26
Lactate, mmol/L	7.0	0.5-1.6
INR	2.48	<1.2
APTT, sec	135	27.0-38.0
Fibrinogen, mg/dL	130	200.0-400.0
D-dimer, µg/mL	>20.0	0.00-0.50
Hemoglobin, g/dL	15.6	13.0-18.0
MCV, fL	97.5	87.0-103.0
Leukocytes, ×10⁹/L	3.95	4.0-11.0
Neutrophils, %	89.1	53.8-69.8
Lymphocytes, %	9.1	25.3-47.3
Platelets, ×10⁹/L	87	150-400
C-reactive protein, mg/dL	15.12	<0.5
Total protein, g/dL	6.2	6.6-8.7
Serum albumin, g/dL	4.0	3.4-4.8
Sodium, mEq/L	142	135-147
Potassium, mEq/L	4.2	3.7-5.1
Chloride, mEq/L	102	96-106
Calcium, mg/dL	8.9	8.6-10.0
Urea, mg/dL	49	0-50
Creatinine, mg/dL	2.3	0.7-1.2
Total bilirubin, mg/dL	1.0	<1.2
AST, U/L	28	<40
ALT, U/L	18	<41
GGT, U/L	58	10-49
Alkaline phosphatase, U/L	91	40-130
Lactate dehydrogenase, U/L	179	135-225
NT-proBNP, pg/mL	5954	<120
Troponin I, ng/mL	0.028	<0.05

Multiplex PCR testing for SARS-CoV-2, influenza A and B, and respiratory syncytial virus (RSV) was negative. The electrocardiogram demonstrated sinus tachycardia without ST-T wave abnormalities. Chest radiography revealed bilateral patchy infiltrates.

**Figure 2:**
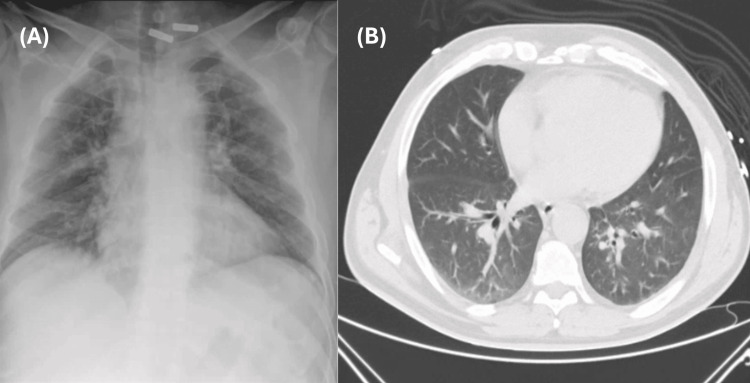
(A) Chest X-ray and (B) thoracic abdominal pelvic CT scan (A) Chest X-ray showing patchy infiltrates. (B) Contrast-enhanced thoraco-abdomino-pelvic CT angiography showing no evidence of pulmonary thromboembolism. The abdominal aorta, celiac trunk, superior mesenteric artery, and renal arteries are patent, with no signs of occlusion and no abnormal abdominal organ findings.

A contrast-enhanced thoraco-abdomino-pelvic CT angiography showed no evidence of pulmonary thromboembolism. The abdominal aorta, celiac trunk, superior mesenteric artery, and renal arteries were patent, with no signs of occlusion or evident aneurysmal dilation at that time. No clear acute abdominal pathology was identified (Figures 2A-2B).

Empiric therapy (within the first 60 minutes of admission) was initiated with intravenous administration of 3 L of normal saline, broad-spectrum antibiotic coverage with piperacillin-tazobactam, and incremental doses of intravenous morphine (total dose: 10 mg). Blood and urine cultures were obtained before the initiation of antibiotics. Blood cultures later yielded growth of *C. canimorsus*. Identification was performed in the hospital microbiology laboratory using routine diagnostic methods, most commonly based on matrix-assisted laser desorption/ionization-time of flight (MALDI-TOF) mass spectrometry. Antimicrobial susceptibility testing was not available.

Despite prompt resuscitative measures, the patient’s condition deteriorated rapidly. He developed worsening metabolic acidosis, hypotension (blood pressure: 60/30 mmHg), tachypnea, and a progressive decline in mental status. Vasopressor support with norepinephrine was initiated, and endotracheal intubation was performed for respiratory failure. Despite intensive supportive care, the patient experienced rapid clinical deterioration and was pronounced dead approximately two hours after arrival. A postmortem examination (full autopsy) revealed rupture of an aortic aneurysm as the cause of death. There was no access to the complete autopsy report; therefore, detailed information regarding the exact location, size, and morphological characteristics of the aneurysm, as well as histopathological or microbiological findings, is not available.

## Discussion

*C. canimorsus* was first described in 1976. It is a genus of gram-negative, facultative anaerobic bacteria that belongs to the *Flavobacteriaceae* family. The bacterium can survive as a commensal in the oral cavity of humans and animals [5]. It is commonly transmitted to humans through bites from dogs or cats, though transmission may also occur via scratches, licking, or other forms of close contact.

However, only 68% of *C. canimorsus* bacteremia cases are linked to animal bites or scratches. Infections usually range from mild to fulminant disease, with shock, respiratory distress, and DIC. Most cases of septicemia occur in patients with impaired host defenses due to splenectomy (33%), alcohol abuse (24%), or immunosuppression (5%). However, 40% of septicemia cases occur in patients with no predisposing conditions. Other infections associated with this microorganism are less frequent and include meningitis, endocarditis, arthritis, and mycotic aneurysm [6].

Mycotic aneurysms are an uncommon but potentially life-threatening condition, accounting for approximately 0.6% of all aneurysms. The most frequently identified causative organisms include *Staphylococcus* species, *Streptococcus pneumoniae*, *Enterococcus*, and *Salmonella* species. In particular, gram-negative bacteria are often associated with more aggressive infections, carrying a higher likelihood of rupture and death than gram-positive organisms. Only a small number of cases of mycotic aneurysms due to *C. canimorsus* have been reported [7]. Although anaerobic cultures can yield accurate results, certain strains of *C. canimorsus* may require more than 48 hours to grow under anaerobic conditions; therefore, empirical treatment should be initiated based on clinical suspicion [8].

At present, the standard management of mycotic aneurysms involves prompt diagnosis, appropriate antimicrobial treatment, and surgical intervention [9]. There are no guidelines for treatment related to the type or duration of antibiotic therapy. Isolated strains have shown susceptibility to a wide range of antibiotics. The first line of treatment is β-lactam/β-lactamase inhibitor combinations. *C. canimorsus* is also known to be susceptible to clindamycin, cephalosporins, quinolones, linezolid, and tetracyclines [10]. Despite the favorable in vitro susceptibility of *C. canimorsus*, the severely impaired host defense mechanisms in affected patients must be considered, as the clinical response to treatment may be delayed or inadequate [8].

Both open and endovascular repair play key roles in identifying the causative organism, controlling the infection, and addressing potential ischemia. This case is an example of the rapid and fatal progression of infection associated with aortic aneurysm rupture in an asplenic patient. Although no animal exposure was identified in our patient, a significant risk factor - splenectomy - was present. The patient's clinical course in the emergency room was rapidly progressive, characterized by systemic inflammatory response evolving into multiorgan failure and coagulopathy. Neurological deterioration progressed from restlessness to decreased responsiveness. Laboratory findings revealed elevated creatinine, liver enzyme abnormalities, and thrombocytopenia with high INR, prolonged APTT, elevated D-dimer, and low fibrinogen, supporting DIC. Vasopressor and respiratory support were also required. This sums five dysfunctions (neurological, cardiovascular, respiratory, renal, and hematologic). At this point, the SOFA (Sequential Organ Failure Assessment) score increased from 7 points (reflecting hematological dysfunction, renal impairment, and circulatory failure) to 9 points with the development of respiratory failure.

Despite aggressive supportive measures and early initiation of antibiotic therapy, the patient's condition continued to deteriorate. The discrepancy between the initial CTA findings and the postmortem diagnosis may be related to factors such as early or subtle aneurysmal changes not detected at the time of imaging, or rapid progression in the context of severe sepsis. A high index of clinical suspicion is essential, even without all the available imaging and microbiological information, as antibiotic susceptibility data often become available only late in the course of bacteremia.

In this case, due to the high risk of septic shock, escalation to a carbapenem was justified, although initial empirical therapy with piperacillin-tazobactam was appropriate and aligned with current first-line recommendations involving beta-lactam/beta-lactamase inhibitor combinations [10]. This escalation was not performed prior to the patient’s death due to the extremely short clinical course and refractory hemodynamic collapse. Vascular repair might have offered a chance for survival if the aortic aneurysm had been detected on the initial CT scan, but the diagnosis was only made post-mortem. 

## Conclusions

This case highlights the fulminant progression of *C. canimorsus* infection in a splenectomized patient, culminating in the fatal rupture of an aortic aneurysm. While the postmortem findings raised the suspicion of a possible infected (mycotic) aortic aneurysm, definitive pathological and microbiological confirmation of an infectious etiology of the aneurysm is not available. Notably, to our knowledge, there was no reported history of animal exposure, underscoring the importance of maintaining a high index of clinical suspicion in high-risk patients, particularly those with asplenia. Although empirical therapy with β-lactam/β-lactamase inhibitor combinations is recommended, early recognition and eventual surgical intervention remain crucial for survival.

*C. canimorsus* infections should be considered in septic patients with risk factors such as splenectomy, even in the absence of contact with animals. The rapid clinical deterioration observed in this case, together with the occurrence of a fatal vascular complication, emphasizes the need for early diagnosis and a multidisciplinary management approach. However, in the absence of direct pathological confirmation, the causal relationship between the infection and aortic rupture must be interpreted with caution, and the presence of a mycotic aneurysm can only be considered likely rather than definitively established.
